# High Fat Diet Alters Lactation Outcomes: Possible Involvement of Inflammatory and Serotonergic Pathways

**DOI:** 10.1371/journal.pone.0032598

**Published:** 2012-03-05

**Authors:** Laura L. Hernandez, Bernadette E. Grayson, Ekta Yadav, Randy J. Seeley, Nelson D. Horseman

**Affiliations:** 1 Department of Molecular and Cellular Physiology, College of Medicine, University of Cincinnati, Cincinnati, Ohio, United States of America; 2 Department of Dairy Science, University of Wisconsin, Madison, Wisconsin, United States of America; 3 Metabolic Diseases Research Institute, University of Cincinnati, Cincinnati, Ohio, United States of America; Baylor college of Medicine, United States of America

## Abstract

Delay in the onset of lactogenesis has been shown to occur in women who are obese, however the mechanism altered within the mammary gland causing the delay remains unknown. Consumption of high fat diets (HFD) has been previously determined to result decreased litters and litter numbers in rodent models due to a decrease in fertility. We examined the effects of feeding a HFD (60% kcal from fat) diet versus a low-fat diet (LFD; 10% kcal from fat) to female Wistar rats on lactation outcomes. Feeding of HFD diet resulted in increased pup weights compared to pups from LFD fed animals for 4 d post-partum. Lactation was delayed in mothers on HFD but they began to produce copious milk volumes beginning 2 d post-partum, and milk yield was similar to LFD by day 3. Mammary glands collected from lactating animals on HFD diet, displayed a disrupted morphologies, with very few and small alveoli. Consistently, there was a significant decrease in the mRNA expression of milk protein genes, glucose transporter 1 (GLUT1) and keratin 5 (K5), a luminobasal cell marker in the mammary glands of HFD lactating animals. Expression of tryptophan hydroxylase 1 (TPH1), the rate-limiting enzyme in serotonin (5-HT) biosynthesis, and the 5-HT_7_ receptor (HTR7), which regulates mammary gland involution, were significantly increased in mammary glands of HFD animals. Additionally, we saw elevation of the inflammatory markers interleukin-6 (IL-6) and tumor necrosis factor-α (TNF- α). These results indicate that consumption of HFD impairs mammary parenchymal tissue and impedes its ability to synthesize and secrete milk, possibly through an increase in 5-HT production within the mammary gland leading to an inflammatory process.

## Introduction

Obesity has profound negative impacts on numerous physiological processes. Prevalence of obesity of adult women in the United States from 1999–2008 is approximately 36%, with approximately 60% of women of a reproductive age (20–39 years of age) being either overweight or obese [1]. Offspring from obese mothers have consistently displayed negative outcomes such as increased birth weights and increased probability of obesity and metabolic syndrome in their lifetime [2]. Furthermore, excessive weight gain during pregnancy increases the mother's risk of developing breast cancer [3]. While much research has been directed towards examining the impact of maternal obesity on the offspring, little has focused on the mammary gland structure itself. Several studies in humans have demonstrated that obese women have a delay (>72 h post-partum) in the arrival of a copious milk supply [4]–[7]. Delayed lactogenesis (failure to lactate for >72 h post-partum) is correlated with a shorter duration of breast-feeding [8]. Other mammalian species also display lactation defects due to obesity/over-feeding. In fact, in dairy cattle over-feeding during the pre-pubertal period has been determined to cause a permanent decrease in milk yield potential [9]. Furthermore, in studies in rat models, pre-pregnancy overweight and obesity has been determined to decrease the response to suckling induced prolactin and disrupt normal mammary gland development during pregnancy [10]. Additionally, consumption of high-fat diets during pregnancy has also been determined to decrease the amount of myoepithelium in the mammary gland, which is thought to increase breast cancer risk [11].

Several studies have been conducted in different mammalian species demonstrating an association with obesity and mammary development during pregnancy. In a study in obese mice, a delay in lactogenesis appeared to be related to the accumulation of lipid droplets within the epithelial cells, and resulted in decreases in milk protein gene expression [12]. In an experiment conducted in gilts, it was determined that feeding of increased energy during the late gestation period resulted in a 27% decrease of total mammary parenchymal weight compared to gilts fed a diet of adequate energy [13]. Diet-induced obesity has also been determined to negatively impact the stromal compartment of the mammary gland and is associated with an increase in breast cancer risk [11].

Obesity is characterized by the activation of the inflammatory process in metabolically active organs in the body. Typically this response leads to the elevation of pro-inflammatory cytokines, adipokines and other inflammatory makers [14]. Recently it was demonstrated that mice consuming HFD exhibit elevated levels of the monoamine 5-HT in serum [15]. It has been suggested that 5-HT is an important mediator of inflammatory responses, including obesity [15]–[17]. In fact, adipocytes have been determined to synthesize and secrete 5-HT [18]. Additionally, in a mouse model of colitis, an inflammatory disease, animals lacking the rate-limiting enzyme for 5-HT synthesis (TPH1) displayed decreased severity of the disease, along with significantly lower macroscopic and histologic damage scores [17]. When animals were then supplemented with 5-hydroxytryptophan, a precursor to 5-HT synthesis that bypasses TPH1, the severity of the colitis was increased and pro-inflammatory cytokines were induced. Specifically, IL-6 and TNF- α were elevated in animals receiving supplemental 5-hydroxy-L-tryptophan, and were decreased in mice lacking TPH1 [17]. Furthermore, serum 5-HT levels were determined to be elevated in mice consuming HFD [15]. This suggests that 5-HT, along with numerous other factors, is in part responsible for the inflammatory process associated with obesity.

Mammary gland involution, like obesity and colitis, resembles an inflammatory process [19]–[21]. In fact, in several species elevated mRNA expression for IL-6 and TNF-α, among other inflammatory markers, in the mammary gland is typical during the involution process [20]–[21]. Serotonin has been previously demonstrated to be responsible for mammary gland involution in several mammalian species [22]–[25]. In the mammary gland, 5-HT accelerates involution in response to milk stasis by acting on the HTR7 to disrupt tight junctions (TJ), as evidenced by decreases in transepithelial resistance as well as decreased levels of tight junction proteins [23], [25]. Transiently, 5-HT acts to decrease TJ permeability through protein kinase A, and sustained increases in 5-HT results in increased TJ permeability by activation of p38 MAP kinase, thereby initiating mammary gland involution [25].

Currently, little is known about the specific processes that are altered within the mammary gland parenchymal tissue in the obese state and how they contribute to the impaired lactation observed in obese subjects. The objectives of this study were to examine the effects of feeding a HFD on the mammary gland and the ability to lactate. Specifically, we also aimed to delineate the involvement of 5-HT in regulating the mammary gland's response to HFD.

## Results

### Pre-pregnancy feed intake and body weight gain

**Figure 1:**
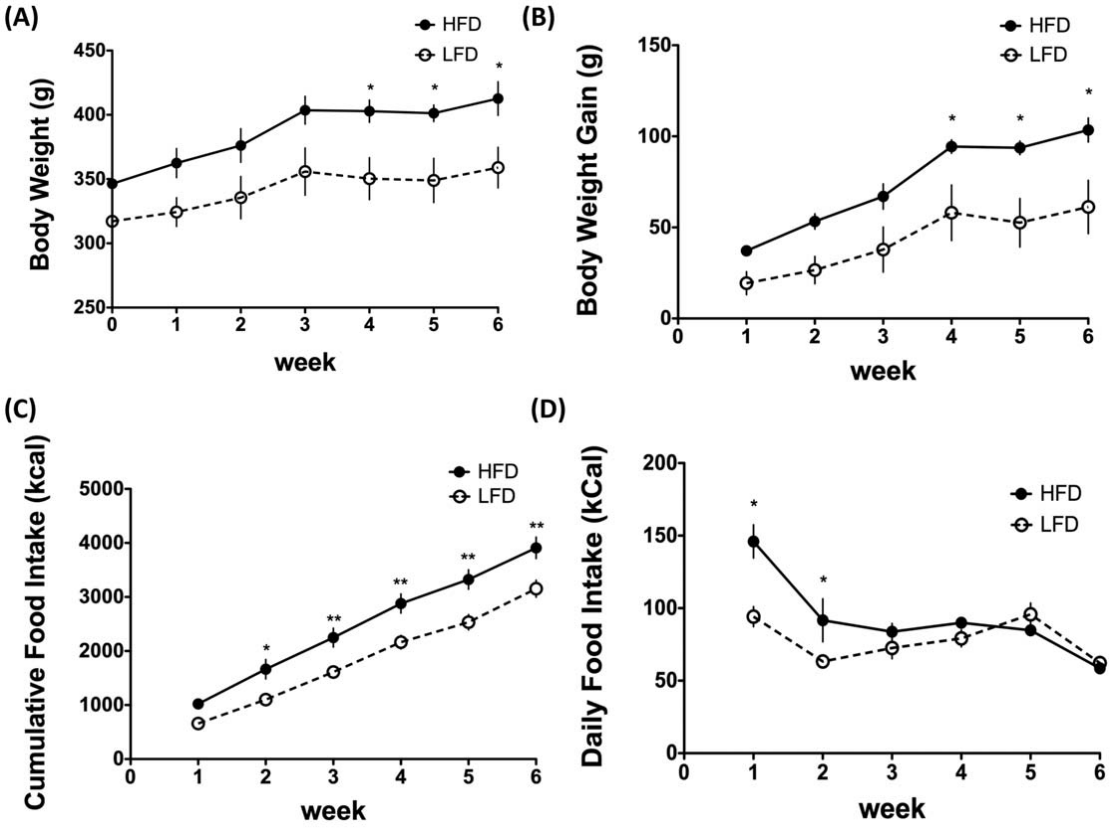
Pre-pregnancy body weights and feed intake in female Wistar rats consuming a HFD vs. LFD. (A) Pre-pregnancy body weights for 6 weeks prior to mating in female Wistar rats consuming a high-fat diet (N = 6; HFD; 60% kcal from fat) versus a low-fat diet (N = 6; LFD; 10% kcal from fat); (B) Pre-pregnancy body weight gain for 6 weeks prior to mating in female Wistar rats consuming a HFD versus a LFD; (C) Pre-pregnancy cumulative food intake for 6 weeks prior to mating in female Wistar rats consuming HFD versus LFD; (D) Pre-pregnancy average daily kilocalorie intake in female Wistar rats consuming HFD versus LFD. Data are represented as mean ± SEM.

HFD animals had significantly higher weekly body weights and body weight gain for the final 3 weeks prior to mating (Figures 1a, b). Cumulative caloric intake was significantly higher in HFD animals during the feeding period pre-mating (Figure 1c). Daily food intake was higher for the first two weeks of feeding in the HFD animals, but did not differ from the LFD animals during the final 4 weeks prior to mating (Figure 1d). Feed intake and body weights were not measured in pregnant and post-partum animals.

### Milk production and pup growth rate

**Figure 2:**
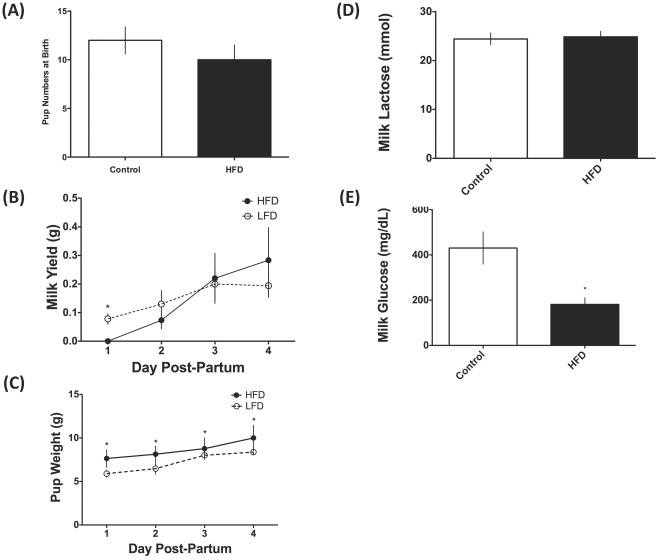
Consumption of HFD alters milk production and pup growth in rats. (A) Milk weights collected by the weigh-suckle-weigh method in female Wistar rats consuming a high-fat diet (HFD; 60% kcal from fat) versus a low-fat diet (LFD; 10% kcal from fat) for 4 d post-partum. (B) Pup weights measured daily for 4 d postpartum in female Wistar rats consuming a high-fat diet (N = 3; HFD; 60% kcal from fat) versus a low-fat diet (N = 6; LFD; 10% kcal from fat). All data are represented as mean ± SEM (* P<0.05).

Both the control (12±1.4) and HFD (10±1.5) cohorts that successfully gave birth did not have statistically different litter sizes (Figure 2a). HFD animals that successfully carried their pregnancies to term failed to produce any measurable milk yield during the weigh-suckle-weigh test on d1 post-partum, while LFD animals were producing approximately 0.1 g of milk during a bout of nursing on d1 post-partum (Figure 2b). Additionally, we attempted to milk all animals and HFD mothers were unable to produce any milk on d 1 post-partum. We also measured PRL and PRL receptor mRNA concentrations in the mammary glands and did not see any significant differences between cohorts, indicating suckling-induced PRL release was consistent between dams from both the LFD-lactating and HFD-lactating groups (data not shown). Beginning d 2 post-partum, HFD animals began producing milk and the volume did not significantly differ from that of LFD animals through 4d post-partum (Figure 2b). Pups from mothers receiving the HFD were significantly heavier than LFD pups at their first weighing, and for the first 4d post-partum (Figure 2c).

Milk lactose was not significantly different between LFD and HFD lactating animals on d 4 of lactation. (Figure 2d). However, milk glucose levels were significantly different between the LFD and HFD cohorts on d 4 lactation (P<0.05; Figure 2e).

### Mammary gland histology and alveolar cells

**Figure 3:**
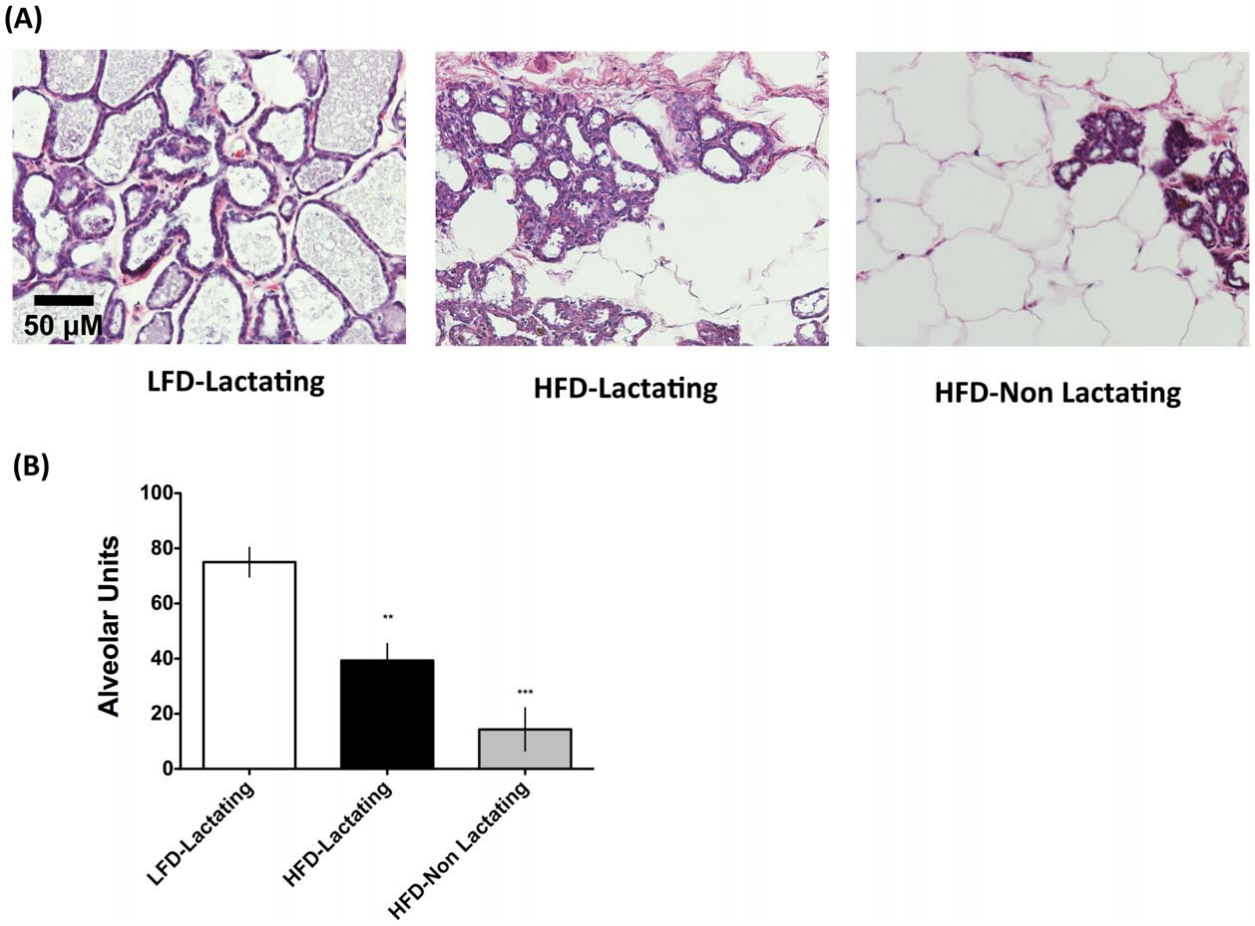
HFD decreases intact alveolar units in the mammary gland of lactating and non-lactating rats. (A) Hemaxtoxylin and eosin stained sections of mammary gland #4 collected from LFD-lactating, HFD-lactating, and HFD-non lactating female Wistar rats on 4 d post-partum at 20× magnifcation. (B) Alveolar units present in LFD-lactating (N = 6), HFD-lactating (N = 3), and HFD-non lactating (N = 3) animals. Number of alveolar units were counted per animal in a field of view at 20× magnification. Data are represented as mean ± SEM (**P<0.01; ***P<0.001).

Lactating animals on the HFD displayed disrupted mammary gland morphologies compared to those on the LFD when examined on d 4 lactation (Figure 3). Lactating-HFD mammary gland tissue contained more and larger adipocytes. The alveolar lumens in the parenchymal tissue were smaller, and the alveoli were irregular, with areas in which the parenchymal tissue appeared to have been deteriorating. Additionally, of the alveoli that were intact, they appeared to mostly be empty, and of the ones that did contain fluid in the luminal space, they were more distended than those in the LFD group. Quantitatively, the number of intact alveolar units were significantly reduced in both the HFD-lactating and HFD-non lactating (post-partum) animals compared to LFD-lactating animals (Figure 3b). While it is not surprising that non-lactating animals had few intact alveolar units, the decrease in intact alveolar units in the HFD-lactating animals suggests that obesity altered the development of the alveolar units necessary for milk production.

### Mammary gene expression

**Figure 4:**
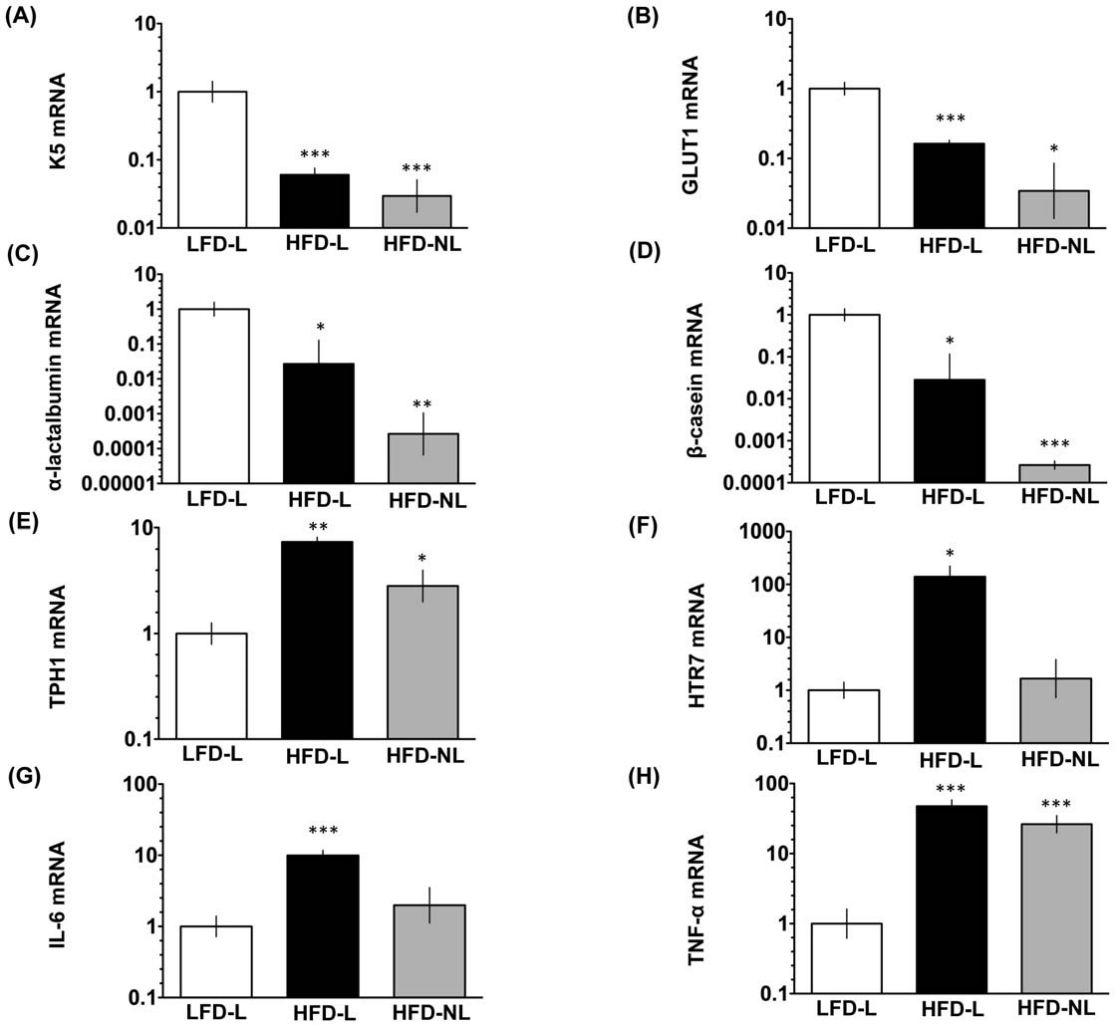
HFD consumption pre-pregnancy alters expression of genes in the mammary gland related to milk synthesis. mRNA expression measured by real-time quantitative PCR for (A) K5, (B) GLUT1, (C) α-lactalbumin, (D) β-casein, (E) TPH1, (F) HTR7, (G) IL-6, and (H) TNF-α in mammary glands collected from LFD-lactating (N = 6), HFD-lactating (N = 3), and HFD-non lactating (N = 3) female Wistar rats. All data was analyzed according to the 2^−ΔΔCt^ method [46]. Data are represented as means ± SEM of the log transformation (*P<0.05; **P<0.01; ***P<0.001).

Gene expression of K5 (a luminobasal cell marker), α-lactalbumin (milk protein gene important in regulation of milk volume), β-casein (milk protein gene), and GLUT1 (glucose transporter important for uptake of glucose in the mammary gland) were significantly depressed in the mammary glands of HFD-lactating and HFD-non lactating animals compared to animals on the LFD (Figures 4a, b, c, d). TPH1 mRNA (rate-limiting enzyme in 5-HT biosynthesis) was significantly increased in HFD-lactating animals compared to LFD-lactating animals (Figure 4e). TPH1 expression in HFD-non lactating animals was intermediate between the two lactating groups. In addition to elevated TPH1 expression, we demonstrated an increase in HTR7 mRNA expression in the HFD-lactating animals compared to LFD-lactating and HFD-non lactating animals (Figure 4f). Mammary transcripts for the inflammatory cytokines IL-6 were elevated in the HFD-lactating cohort and TNF-α in both the HFD-lactating and HFD-non lactating cohorts (Figures 4g, h).

## Discussion

Obesity in humans and animals has been determined to contribute to disruption of normal lactation cycles in mothers. Several studies have indicated that maternal obesity contributes to delayed onset of lactogenesis, reduced breast-feeding duration, as well increased birth weights [6], [26]–[27]. Over-nutrition and obesity in animal models have also been implicated in decreased milk yield potential and lactogenesis, and have negative impacts on mammary gland development during pregnancy [9], [12]–[13], [28]–[29]. In this paper we show that consumption of a HFD results in lactation defects that are directly related to alteration of the parenchymal tissue of the mammary gland during lactation and are associated with evidence for increased inflammation and activity of 5-HT systems. Additionally, we confirm that consumption of HFD in female rats decreased fertility of the animals, as well as increased weights of the offspring, which have been reported in numerous other studies [2], [30]–[34].

In our study, we demonstrate that the mammary glands of rats consuming HFD had a dramatic decrease in the number of intact alveolar units within the gland, which are necessary for lactogenesis. Unlike the previously mentioned study [12], we did not see excessive lipid droplet accumulation within the alveolar units. Furthermore, we demonstrated a decrease in the marker for luminobasal (glandular and myoepithelial) epithelial cells, K5 [35]–[36]. K5 is a known marker of for both the glandular epithelial and myoepithelial cells in the mammary gland and its presence in breast cancer epithelial cells is typical of triple-negative breast tumors [36]. While animals consuming the HFD had greatly reduced milk yield on d 1 post-partum, they regain the ability to lactate for the remaining three days we measured milk volumes. Our results confirm studies in humans that demonstrate a delay in the onset of lactogenesis in obese women, but not a complete loss of the ability to lactate for the entire lactation cycle [6]. The fact that pups did not decrease in body weight on the first day of lactation is indicative of studies that demonstrate an increased body weight at birth in obese mothers [6], [26]–[27]. Had dams not recovered their milk production after this date, we would have expected pup weights to decrease. This indicates that feeding of HFD is responsible for the deterioration of the alveolar units necessary for milk synthesis.

Lactogenesis is regulated by a variety of factors. It has been previously demonstrated that consumption of HFD prior to pregnancy negatively alters the mammary gland developmental process that occurs during pregnancy [12], [37]–[39]. Little is known about the impact that HFD consumption has on the mammary gland during lactation. Glucose transport into the mammary gland occurs through the GLUT1 transporter [40]. This is a critical step in the synthesis of lactose, the primary carbohydrate and osmotic constituent of milk. GLUT1 is responsible for the delivery of glucose to the site of lactose synthesis [40]–[41]. Lactose is critical to human infants in particular in order to aid in temperature regulation by providing a large amount of free water to the neonate [42]. We demonstrate that HFD results in decreased mRNA expression of GLUT1 in the mammary gland of lactating rats compared to LFD animals. This suggests that one consequence of over-nutrition in the mammary gland is decreased ability to take up glucose for lactose synthesis through the GLUT1. Furthermore, we saw a decrease in milk glucose levels on d 4 of lactation in HFD dams, however we saw no differences in milk lactose between cohorts. In addition to a significant decrease in GLUT1 mRNA and milk glucose, we also saw significant decreases in the major milk protein genes α-lactalbumin and β-casein. α-lactalbumin is critical in the formation of lactose from glucose and galactose and β-casein along with other types of caseins, are important for calcium binding and growth of the offspring [42].

In addition to investigating the effects of HFD on synthesis of lactose and milk proteins in the mammary gland, we determined that TPH1 and HTR7 expression were increased in the mammary glands of lactating HFD animals compared to LFD animals. TPH1 is the rate-limiting enzyme in 5-HT biosynthesis, which has been previously reported to accelerate involution in the mammary gland via an HTR7 mediated pathway [22]. Elevation of TPH1 expression in the mammary gland of lactating HFD animals is a potential explanation for the decreased levels of alveolar units and secretory epithelium seen in the mammary glands of these animals. Additionally, 5-HT has been demonstrated to accelerate mammary gland involution through disruption of tight junctions between the epithelial cells through stimulation of an HTR7 signaling pathway [33]. Furthermore, the existence of a 5-HT signaling system in adipocytes has been reported [18]. The increased number of adipocytes in the HFD-lactating animals, could be an additional source of 5-HT. In addition to elevated 5-HT synthesis within the mammary gland of HFD-lactating animals, as well as increased HTR7 expression, substantial increases in expression of two key pro-inflammatory cytokines, IL-6 and TNF-α, were seen. Mammary gland involution has often been likened to an inflammatory process [20]. Furthermore, obesity is often responsible for the activation of inflammatory processes in metabolically active sites in the body [43]. Both mammary gland involution and obesity have been demonstrated to result in the elevation of a variety of inflammatory regulators, such as IL-6 and TNF-α, among others [14], [20]–[21], [43]. Specifically, mammary gland levels of IL-6 and TNF- α are relatively low until the onset of involution [19], [20]. IL-6 has been determined to regulate mammary gland involution independent of the signal transducer and activation of transcription 3 (Stat3) pathway, whereas TNF- α activates the Stat3 pathway [19]–[20], [34]. This suggests that these cytokines are working through different signaling mechanisms due to HFD to induce mammary gland involution, thereby supporting the lack of IL-6 mRNA expression in the non-lactating group. Additionally, in skin epithelium, decreased expression of K5 has been demonstrated to result in increased IL-6 expression [44]. Serotonin has been described as a mediator of the pathogenesis of inflammation in the condition of experimental colitis [17]. Recently, it was also reported that 5-HT serum levels are elevated in mice consuming HFD [15]. It is plausible that consumption of HFD lead to the stimulation of the 5-HT pathway in the mammary gland leading to an inflammatory-involution process that disrupted the parenchymal tissue. This is supported by the increased expression of the pro-inflammatory cytokines IL-6 and TNF-α in the mammary gland of HFD-lactating rats.

In conclusion, we demonstrate that feeding of HFD results in complications for maintaining pregnancy, delayed lactogenesis, increased offspring weights, and disrupted mammary gland development, possibly due to an inflammatory process within the gland. Additionally, we demonstrate that within the mammary gland itself, there is a depression in genes associated with uptake of glucose and formation of milk proteins and increases in genes associated with the inflammatory process. Additionally, we demonstrate a decrease in milk glucose levels in HFD animals. Furthermore, we demonstrate a decrease in the transcript for luminobasal cells, which are important to the differentiation of the mammary gland. We also demonstrate that the mammary gland of HFD animals have an increased capacity to produce 5-HT which has previously been shown to have a negative impact on the secretory epithelium and to be a potent stimulator of inflammation. Future research should be directed toward determining the contribution of HFD consumption to stimulation of 5-HT mediated inflammatory processes in the mammary gland. Furthermore, determination of the effects of HFD on other components of milk composition should be determined.

## Materials and Methods

### Ethics Statement

The animal experiments were approved by the Institutional Animal Care and Use Committee at the University of Cincinnati and their guidelines for the care and use of the animals were strictly followed for all experiments. The protocol number assigned for these experiments was 05-01-11-01 entitled Hormone and Genetic Studies in Rodent.

### Animal care and experiment design

These experiments were approved by the Institutional Animal Care and Use Committee at the University of Cincinnati. Female Wistar Rats (300–350 g) purchased from Harlan Laboratories and were housed individually and the Metabolic Diseases Research Institute, University of Cincinnati, at 25°C, constant humidity (50–60%), and a 12 hr light/dark cycle with free access to food and water. Animals were randomly assigned to a HFD ([45]; #D12492, Research Diets Inc., 60% fat; 5.24 kcal/g; n = 6) or LFD ([45]; #D12450B, Research Diets Inc.; 10% fat; 3.85 kcal/g; n = 6) for 6 weeks prior to mating and were given free access to food and water. Females were mated, and all 6 LFD animals conceived and achieved lactation, while only 3 of the HFD animals successfully gave birth. For breeding purposes, female rats were moved to the male home cage, male∶female ratio, 1∶1 for 5 days weekly and then separated until successful breeding occurred. Animals were then permanently separated for the remainder of gestation.

Beginning on the day of parturition, and continuing for 4 d post-partum, animals were subjected to a weigh-suckle-weigh experiment in which pups were removed from their mothers for 4 h at 0800 h and, weighed, and then at 1200 h returned to their mothers to nurse for 30 min, and weighed again to estimate milk yield. Litter sizes were standardized to 10 pups/dam for all animals to ensure equal sucking intensity and milk removal from the dam. In addition, to confirm the presence of milk in the glands, we milked all dams during the 4 d post-partum. Animals were give 0.4 units of oxytocin to induce milk ejection, and milk was collected by vacuum pump. Furthermore, pups were weighed daily for 4 d post-partum. Milk yield and pup weights are reported on a pup basis. On d 4 post-partum, animals were sacrificed and mammary glands were collected for RNA and histology. Three HFD animals were unable to successfully deliver pups and lactate. These animals were also sacrificed and mammary glands were extracted for RNA and histology.

### Histology

The #4 mammary gland was collected from 4 d post-partum LFD lactating (n = 6), HFD lactating (n = 3), and non-pregnant HFD animals (n = 3). Tissues were fixed in 4% paraformaldehyde overnight at 4°C and were embedded and stained for hematoxylin and eosin. Alveolar units were quantified in the #4 mammary gland for each animal and images were taken at a magnification of 20×.

### Real-Time Quantitative RT-PCR

Total RNA was extracted from the mammary glands of LFD lactating, HFD lactating, and HFD non-pregnant rats using TRIreagent (Molecular Research Products). mRNA was reverse-transcribed using the QuantiTect Reverse Transcription Kit (Qiagen). Genes examined included the following: K5- forward 5′-GTTACAGAGCCACCCACAGC-3′, reverse 5′-AGAGACAGATGGGGTGATGG -3′; GLUT1- forward 5′-GTGGGCCTCTTTGTTAATCG-3′, reverse 5′-CACATACATGGGCACAAAGC-3′; TPH1- forward 5′-GAACTCCAGTGGCTTTGAGG-3′, reverse 5′-ACAGGTTCACGTGGTTCTCC-3′, HTR7- forward 5′-GTGGCTTCCTAGAGGTGACG-3′, reverse 5′-TGAGGTCGGTGACACTAACG-3′; α-lactalbumin- forward 5′- ATGGCTATCAAGGCATCAGC-3′, reverse- 5′-CAGCTTCTCAGAGCACATGG-3′; β-casein- forward 5′- CGCATAAGGCTTCAACTTGC-3′, reverse-5′- AATGGGACTGCAAGAGATGG-3′; IL-6- forward 5′-GATGGATGCTTCCAAACTGG-3′, reverse- 5′-AGGAGAGCATTGGAAGTTGG-3′; TNF-α-forward 5′-ACGATGCTCAGAAACACACG-3′, reverse- 5′-CAGTCTGGGAAGCTCTGAGG-3′; prolactin-forward 5′-TCTGTTCTGCCAAAATGTGC-3′, reverse-5′-AGGAGTGCACCAAACTGAGG-3′; prolactin receptor-forward 5′ ACAGAGCTCACGTCCTTTGC-3′, reverse-5′-ACCAGCAGGTGAATGTTTCC-3′; ribosomal S15- forward-5′- CTTCCGCAAGTTCACCTACC-3′, reverse-5′-GTTGTACACACCCACCATGC-3′. Primer specificity was assessed by the presence of a single temperature dissociation peak. Ribosomal S15 was used as the housekeeping gene and analysis was conducted using the 2^−ΔΔCt^ method [46].

### Milk Glucose and Lactose

Milk glucose was analyzed using the methods of Karkalis [47]. Milk lactose was analyzed in milk collected from animals on d 4 of lactation using a Biovision lactose analysis kit #K624, per manufacturer's instructions (Biovision).

### Statistical Analysis

Data are expressed as mean ± SEM. Statistical analysis was conducted using Prism for Macintosh, version 5.0d (GraphPad Software). Pup weights and milk yield were analyzed using a two-way ANOVA followed by Bonferroni's post-hoc test for multiple pairwise comparisons. Alveolar units were analyzed using a one-way ANOVA followed by a Tukey's post-hoc test for multiple pairwise comparisons. Relative gene expression as calculated using the 2^−ΔΔCt^ method was analyzed using a one-way ANOVA followed by a Tukey's post-hoc test for multiple pairwise comparisons.
